# A global synthesis of high-resolution stable isotope data from benthic foraminifera of the last deglaciation

**DOI:** 10.1038/s41597-023-02024-2

**Published:** 2023-03-10

**Authors:** Juan Muglia, Stefan Mulitza, Janne Repschläger, Andreas Schmittner, Lester Lembke-Jene, Lorraine Lisiecki, Alan Mix, Rajeev Saraswat, Elizabeth Sikes, Claire Waelbroeck, Julia Gottschalk, Jörg Lippold, David Lund, Gema Martinez-Mendez, Elisabeth Michel, Francesco Muschitiello, Sushant Naik, Yusuke Okazaki, Lowell Stott, Antje Voelker, Ning Zhao

**Affiliations:** 1 Centro para el Estudio de los Sistemas Marinos, CONICET, 2915 Boulevard Brown, U9120ACD Puerto Madryn, Argentina; 2 MARUM – Center for Marine Environmental Sciences, University of Bremen, Bremen, Germany; 3 Department of Climate Geochemistry, Max Planck Institute for Chemistry, Hahn-Meitner Weg 1, 55128 Mainz, Germany; 4 College of Earth, Ocean, and Atmospheric Sciences, Oregon State University, Corvallis, OR USA; 5 Alfred Wegener Institute Helmholtz Centre for Polar and Marine Research, Bremerhaven, Germany; 6 Department of Earth Science, University of California, Santa Barbara, CA 93106 USA; 7 Micropaleontology Laboratory, Geological Oceanography Division, National Institute of Oceanography, Goa, India; 8 Department of Marine and Coastal Sciences, Rutgers University, New Brunswick, NJ USA; 9 LOCEAN/IPSL, Sorbonne Université-CNRS-IRD-MNHN, UMR7159 Paris, France; 10 Institute of Geosciences, Kiel University, Kiel, Germany; 11 Institute of Earth Sciences, Heidelberg University, Heidelberg, Germany; 12 Department of Marine Sciences, University of Connecticut - Avery Point, Groton, CT 06340 USA; 13 Helmholtz Institute for Functional Marine Biodiversity at the University of Oldenburg (HIFMB), Ammerländer Heerstrasse 231, D-26129 Oldenburg, Germany; 14 LSCE-IPSL (CEA-CNRS-UVSQ), Paris-Saclay University, 91190 Gif-sur Yvette, France; 15 Department of Geography, University of Cambridge, Cambridge, CB2 3EQ UK; 16 Centre for Climate Repair at Cambridge, Downing College, Cambridge, CB2 1DQ UK; 17 CSIR-National Institute of Oceanography, Dona Paula, Goa India; 18 Graduate School of Science, Kyushu University, Nishi-ku, Fukuoka, 819-0395 Japan; 19 Department of Earth Sciences, University of Southern California, Los Angeles, CA USA; 20 Instituto Portuguēs do Mar e da Atmosfera, Divisâo de Geologia e Georecursos Marinhos, Av. Doutor Alfredo Magalhâes Ramalho 6, 1495-165 Alges, Portugal; 21 Centre of Marine Sciences, Universidade do Algarve, Faro, Portugal; 22 State Key Laboratory of Estuarine and Coastal Research & School of Marine Science, East China Normal University, Dongchuan Rd 500, 200241 Shanghai, China

**Keywords:** Palaeoceanography, Marine chemistry

## Abstract

We present the first version of the Ocean Circulation and Carbon Cycling (OC3) working group database, of oxygen and carbon stable isotope ratios from benthic foraminifera in deep ocean sediment cores from the Last Glacial Maximum (LGM, 23-19 ky) to the Holocene (<10 ky) with a particular focus on the early last deglaciation (19-15 ky BP). It includes 287 globally distributed coring sites, with metadata, isotopic and chronostratigraphic information, and age models. A quality check was performed for all data and age models, and sites with at least millennial resolution were preferred. Deep water mass structure as well as differences between the early deglaciation and LGM are captured by the data, even though its coverage is still sparse in many regions. We find high correlations among time series calculated with different age models at sites that allow such analysis. The database provides a useful dynamical approach to map physical and biogeochemical changes of the ocean throughout the last deglaciation.

## Background & Summary

The stable isotopic ratio of carbon and oxygen of benthic foraminifera, commonly expressed in delta notations (*δ*^13^C and *δ*^18^O) when compared with the ratio of established standards, are often used as tracers of ocean circulation, climate and carbon cycle processes. *δ*^18^O values from CaCO_3_ tests of epibenthic to shallow infaunal foramifera have been linked to bottom water temperatures and sea level^1,2^, sea water densities^3^, transport rates^4–6^ as well as the transport in the deep ocean^7^. The *δ*^13^C values from CaCO_3_ tests traces the *δ*^13^C values of bottom water dissolved inorganic carbon (DIC) and is used to infer carbon cycling and the distribution of deep ocean water masses^8–11^.

Despite the relatively large amounts of existing data, the use of stable isotope compilations in paleoclimate research is hindered by the following issues:

- Heterogeneous, dispersed data: Data from sediment cores are typically processed, analyzed, and archived separately in data repositories or personal computers. The format and content of the data files varies across cores and operators, and often different data files for a single core exist. Thus, paleoceanographic data in existing repositories are highly heterogeneous. This makes compiling data difficult and time-consuming, complicating their reusability.
- Age models: Interpretations of paleoceanographic data require age-depth models to associate the depths in core with calendar ages. Different types of age constraints exist, for instance ^14^C dates^12^, ash layers^13^, alignment to benthic or planktonic foraminiferal *δ*^18^O variations^14^, surface temperatures, magnetic properties^15^ or ^14^C features^16^. Additionally, multiple age models can be produced from the same underlying age data depending on the software package used, adjustable parameters within the software package, the atmospheric radiocarbon calibration curve used, and the radiocarbon reservoir ages assumed for the core site. The diversity of methodologies makes it difficult to compare stable isotope time series from cores provided by different sources, especially for climate change events such as during the last deglaciation (~20-10 thousand years before present (ky BP)).
- Species offsets: Because of its epifaunal (i.e., on and slightly above the sea floor) habitat, *δ*^13^C determined from tests of the genus *Cibicidoides*, in particular *Cibicidoides wuellerstorfi*, has the lowest offsets with respect to *δ*^13^C of DIC^11^, making it the preferred analyzed species for *δ*^13^C values of seawater DIC reconstructions. However, numerous sites include *δ*^13^C values determined from other species or even genera, including infaunal *Uvigerina*, which yield higher offsets. Benthic foraminiferal *δ*^18^O values are also affected by species offsets^17^, and some publications include species-specific corrections to obtain equilibrium or seawater *δ*^18^O^18^.

The Ocean Circulation and Carbon Cycling (OC3) working group of the Past Global Changes (PAGES) program seeks to understand global ocean carbon cycling, ocean circulation and climate during the last deglaciation. One major goal is to create a global database of *δ*^13^C and *δ*^18^O data from benthic foraminifera that would overcome the shortcomings outlined above. OC3 members have developed specific targets, criteria for inclusion of data, a quality control procedure, and a database structure. One of the specific goals is that the new database should be easy to update in the future and extendable to other variables. Specifically, the OC3 database is an ever-evolving database that can be used for many different purposes beyond the specific scientific goals of OC3. Its first version, which is presented here, consists of a compilation of high-resolution benthic foraminiferal *δ*^13^C and *δ*^18^O time series from the global ocean. Stable isotopes of oxygen and carbon of benthic foraminifera as well as data used for the calculation of age models are compiled, including different age models for each site, when available. All components undergo a quality control to standardize the database, and we only include sites that can resolve millennial-scale changes associated with the last deglaciation.

One important goal of OC3 is to quantify uncertainty. This includes chronostratigraphic uncertainties. For this purpose, we included different age models for sediment cores, if multiple age model approaches are available. The OC3 database archives both stable isotope data and age model information, yet separately. In other words, isotope data are kept separate from age model information, but a connection of both is provided by the OC3 database. This facilitates future updates of age models without information loss. When available, the database includes all relevant data necessary to construct the age model, such as radiocarbon dates, reservoir age corrections, and tie points to reference records.

The purpose of this paper is to describe the first version of the OC3 database. We describe its structure and list the sites and age models included. We then describe several programming tools used to facilitate analysis of the database. Finally, we illustrate the utility of the database by comparing different age models across the last deglaciation.

## Methods

### Data acquisition

Benthic foraminiferal *δ*^13^C and *δ*^18^O data from global marine sediment core sites were collected from on-line repositories, original publications, personal communications, and recent data compilations (Tables 1–6). Species included in the database are displayed in Table 7. We include benthic foraminiferal species from the genus *Cibicidoides*, especially *Cibicidoides wuellerstorfi*. Some *Uvigerina* stable isotope data are also included, in particular for the sake of documentation of previously-unpublished sites. We define a data quality control protocol to identify “good data”, of sufficient quality and resolution according to the following criteria:

- The temporal resolution of the benthic foraminiferal *δ*^13^C and/or *δ*^18^O data is 1 ky or better for the Last Glacial Maximum (LGM, 23-19 ky BP) and/or early deglaciation (ED, 19-15 ky BP).
- The original publication, as well as the source of the isotope data and age models, were checked for differences with the values presented in the database. When possible, a quality control was performed by the original author or compiler of the data. Data sources labeled as personal communications were provided directly from the original owner of the data to the authors of this work.
- We identified whether species-specific corrections were applied to the raw stable isotope data. Both uncorrected and corrected data are reported in the database.
- Outliers and hiatuses, when reported in the original publications, were checked for and marked.
- Species names were checked and standardized within the database.

**Table 1:** Sites from the OC3 deglacial compilation.

Site	Latitude (°N)	Longitude (°E)	Depth (m)	Isotope data reference	Age models
**ATLANTIC OCEAN**					
ALB226	17.95	−21.05	3100	Sarnthein *et al*.^40^	OC320
BOFS14K	58.63	−19.43	1756	Bertram *et al*.^41^	J + R; OC320
BOFS17K	58	−16.5	1150	Shimmield *et al*.^42^	J + R
BOFS26-6k	24.45	−19.84	3680	Beveridge *et al*.^43^	J + R
BOFS28-3K	24.61	−22.76	4900	Beveridge *et al*.^43^	J + R
BOFS29-1K	20.52	−21.12	4000	Beveridge *et al*.^43^	J + R
BOFS30_1K	19.74	−20.72	3580	Beveridge *et al*.^43^	OC320
BOFS31_1K	19.00	−20.16	3300	Beveridge *et al*.^43^	OC320
CD154-10-06P	−31.17	32.89	3076	Simon *et al*.^44^	OC320
CH69-K09	41.76	−47.35	4100	Waelbroeck *et al*.^45^	J + R; OC320; W13; W20
CH73-139	54.63	−16.35	2209	Duplessy *et al*.^46^	J + R; OC320
CH74-227	−35.27	−29.25	3225	Labeyrie *et al*.^47^	J + R; OC320
CH75-04	10	−56	3820	Curry *et al*.^48^	J + R
CH82-20PC	43.5	−29.87	3020	Keigwin *et al*.^49^	J + R; OC320
EW9209-1JPC	5.91	−44.19	4056	Curry *et al*.^48^	J + R; OC320; W13; W20
EW9209-2JPC	5.64	−44.47	3528	Curry *et al*.^50^	J + R; OC320
EW9209-3JPC	5.31	−44.26	3288	Curry *et al*.^50^	J + R; OC320
EW9302-24GGC	62	−21.67	1629	Oppo *et al*.^51^	J + R; OC320
EW9302-25GGC	62.06	−21.47	1523	Oppo *et al*.^51^	OC320
EW9302-26GGC	62.32	−21.46	1450	Oppo *et al*.^51^	OC320
GeoB1105-4	−1.66	−12.43	3225	Bickert *et al*.^52^	J + R; OC320
GeoB1515-1	4.24	−43.67	3129	Vidal *et al*.^53^	OC320
GeoB16202-2	−1.91	−41.59	2248	Voigt *et al*.^54^	J + R; OC320; W20
GeoB16206-1	−1.58	−43.02	1367	Voigt *et al*.^54^	J + R; OC320
GeoB16224-1	6.66	−52.08	2510	Voigt *et al*.^54^	J + R; OC320
GeoB1711	−25.53	12.63	1967	Waelbroeck *et al*.^45^	OC320; W13; W20
GeoB1720-2	−28.99	13.83	1997	Dickson *et al*.^55^	J + R; OC320; W13; W20
GeoB2104-3	−27.28	−46.37	1503	Mulitza *et al*.^56^	OC320
GeoB3004-1	14.60	15.92	1803	Schmiedl *et al*.^57^	OC320
GeoB3104	−3.67	−37.72	767	Arz *et al*.^58^	J + R; P; OC320
GeoB3808-6	−30.81	−14.71	3213	Jonkers *et al*.^59^	J + R; OC320
GeoB4216-1	30.63	−12.4	2324	Freudenthal *et al*.^60^	J + R; OC320
GeoB4240-2	28.89	−13.23	1358	Freudenthal *et al*.^60^	J + R; OC320; W13; W20
GeoB4901-8	2.68	6.72	2184	Zabel *et al*.^61^	J + R
GeoB6408-4	−43.61	−20.44	3797	Mulitza *et al*.^62^	OC320
GeoB6718	52.2	−12.8	900	Dorschel *et al*.^63^	P
GeoB6719-1	52.15	−12.77	758	Ruggeberg *et al*.^64^	OC320
GeoB7010-2	8.57	−53.20	2549	Govin *et al*.^65^	OC320
GeoB7920-2	20.8	−18.6	2278	Tjallingii *et al*.^66^	J + R; P; OC320; W13; W20
GeoB9506-1	15.61	−18.35	2956	Mulitza *et al*.^62^	OC320
GeoB9508-5	15.5	−17.9	2384	Mulitza *et al*.^67^	J + R; P; OC320; W13; W20
GeoB9510-1	15.42	−17.65	1566	Völpel *et al*.^68^	OC320
GeoB9526	12.4	−18.1	3223	Zarriess *et al*.^69^	J + R; P; OC320; W13; W20
GeoB13601-4	12.43	−18.00	2997	Just *et al*.^70^	OC320
GeoB13731-1	35.41	−2.55	362	Fink *et al*.^71^ Wang *et al*.^72^	OC320
GeoB17402-2	8.00	126.57	556	Shao *et al*.^73^	O; OC320
GEOFAR-KF13	37.58	−31.84	2690	Jonkers *et al*.^23^	J + R; OC320; W13; W20
GEOFAR-KF16	38	−31.13	3050	Repschläger *et al*.^74^	OC320; W13; W20
GIK11944-1	35.65	−8.06	1765	Weinelt *et al*.^75^	J + R
GIK12379-3	23.1	−17.8	2136	Sarnthein *et al*.^40^	P
GIK12392-1	25.17	−16.85	2575	Sarnthein *et al*.^40^	OC320; W13; W20
GIK13289-2	18.07	−18.01	2485	Sarnthein *et al*.^40^	J + R; OC320
GIK15612-2	44.36	−26.54	3050	Sarnthein *et al*.^40^	J + R; OC320
GIK15637-1	27	−18.99	3849	Sarnthein *et al*.^40^	J + R; OC320
GIK15666-6	34.9	−7.1	803	Weinelt *et al*.^76^	J + R; P
GIK15669-1	34.89	−7.82	2022	Sarnthein *et al*.^40^	OC320; W13; W20
GIK15670-5	34.91	−7.58	1482	Sarnthein *et al*.^40^	J + R; OC320
GIK16004-1	29.98	−10.65	1512	Sarnthein *et al*.^40^	J + R; P; OC320
GIK16006-1	29.3	−11.5	796	Sarnthein *et al*.^40^	P; OC320
GIK16017	21.3	−17.8	812	Sarnthein *et al*.^40^	P; OC320

Age models citations are listed with letter and number codes: (J + R)^23^ or^18^; (P)^19^; (W13 and W20)^12^, using the IntCal13 or 20 calibration curves, respectively; (OC313) this work, calculated from ^14^C AMS dates using the IntCal13 calibration curve; (OC320) this work, calculated from ^14^C AMS dates using the IntCal20 calibration curve; (M) this work, calculated with *δ*^18^ O alignment; (O) from the original publication (quality checked).

**Table 2:** Continuation of Table 1.

Site	Latitude (°N)	Longitude (°E)	Depth (m)	Isotope data reference	Age models
**ATLANTIC OCEAN**					
GIK16030	21.2	−18.1	1500	Sarnthein *et al*.^40^	P; OC320
GIK16402	14.4	−20.5	4202	Sarnthein *et al*.^40^	P
GIK16415	9.6	−19.1	3841	Sarnthein *et al*.^40^	P
GIK17045-3	52.42	−16.66	3663	Sarnthein *et al*.^40^	J + R
GIK17049-6	55.26	−26.72	3331	Jung *et al*.^77^	J + R; OC320
GIK17050-1	55.47	−27.89	2795	Jung ^78^	J + R
GIK17051	56.2	−31.9	2295	Jonkers *et al*.^23^	J + R; P
GIK23258-2	75	13.97	1768	Sarnthein *et al*.^79^	J + R; OC320
GIK23415-9	53.18	−19.14	2472	Jonkers *et al*.^23^	J + R; OC320; W13; W20
GIK23416-4	51.57	−20	3616	Jung ^78^	J + R
GIK23417-1	50.7	−19.4	3850	Jung *et al*.^77^	P
GIK23418-8	52.6	−20.3	2841	Jung *et al*.^77^	P; OC320
GIK23419-8	54.96	−19.75	1487	Jung ^78^	J + R; P
GIK23519-5	64.8	−29.6	1893	Millo *et al*.^80^	J + R; P; OC320
GL-1090	−24.92	−42.51	2225	Santos *et al*.^81^	OC320; W20
GL-1180	−8.45	−33.55	1037	Nascimento *et al*.^82^	O
GS07-150-17_1GC	−4.22	−37.08	1000	Voigt *et al*.^54^ Freeman *et al*.^83^	O; OC320; W20
IOW226920-3	−22.45	12.36	1683	Mollenhauer *et al*.^84^	OC320
HU-90-013-013P	58.21	−48.37	3380	Hillaire *et al*.^85^	J + R
IODP-303-U1308	49.88	−24.23	3883	Hodell *et al*.^86^	P
KNR110-50GGC	4.87	−43.21	3995	Curry *et al*.^87^	J + R; OC320
KNR110-55GGC	4.95	−42.89	4556	Curry *et al*.^87^	J + R
KNR110-58GGC	4.79	−43.04	4341	Curry *et al*.^87^	J + R
KNR110-66GGC	4.56	−43.38	3547	Curry *et al*.^87^	J + R
KNR110-71GGC	4.36	−43.7	3164	Curry *et al*.^87^	J + R
KNR110-75GGC	4.34	−43.41	3063	Curry *et al*.^87^	J + R
KNR110-82	4.34	−43.49	2816	Curry *et al*.^87^	J + R
KNR140-39GGC	31.67	−75.42	2975	Keigwin *et al*.^88^	OC320
KNR140-51GGC	32.78	−76.28	1790	Keigwin *et al*.^89^	J + R; OC320; W13; W20
KNR159-5-14GGC	−26.68	−46.5	441	Lund *et al*.^34^	OC320
KNR159-5-17JPC	−27.7	−46.49	1627	Lund *et al*.^34^	P; OC320
KNR159-5-20JPC	−28.64	−45.54	2951	Lund *et al*.^34^	P; OC320
KNR159-5-22GGC	−29.78	−45.58	3924	Lund *et al*.^34^	J + R; P; OC320
KNR159-5-30GGC	−28.13	−46.07	2500	Lund *et al*.^34^	P; OC320
KNR159-5-33GGC	−27.57	−46.18	2082	Lund *et al*.^34^	P; OC320
KNR159-5-36GGC	−27.27	−46.47	1268	Oppo *et al*.^90^	J + R; P; OC320; W13; W20
KNR159-5-42JPC	−27.76	−46.63	2296	Lund *et al*.^34^	P; OC320; W13; W20
KNR159-5-54GGC	−29.53	−43.33	4003	Hoffman *et al*.^91^	J + R
KNR159-5-63GGC	−28.36	−45.84	2732	Lund *et al*.^34^	P; OC320
KNR159-5-78GGC	−27.48	−46.33	1829	Lund *et al*.^34^	P
KNR159-5-90GGC	−27.35	−46.63	1105	Lund *et al*.^34^	P; OC320
KNR159-5-125GGC	−29.53	−45.08	3589	Lund *et al*.^34^	J + R; P; OC320
KNR166-2-26JPC	24.33	−83.25	546	Lynch-Stieglitz *et al*.^92^	J + R; OC320; W13; W20
KNR166-2-29JPC	24.28	−83.27	648	Lynch-Stieglitz *et al*.^92^	J + R; OC320; W13; W20
KNR166-2-31JPC	24.22	−83.3	751	Came *et al*.^93^ Came *et al*.^94^ Lynch-Stieglitz *et al*.^92^	J + R; W13; W20
KNR166-2-73GGC	23.74	−79.43	542	Lynch-Stieglitz *et al*.^92^	J + R; OC320; W13; W20
KNR166-2-132JPC	24.85	−79.28	739	Lynch-Stieglitz *et al*.^92^	J + R; OC320
KNR197-10-5GGC	37.09	−31.93	2127	Repschläger *et al*.^18^	J + R
KNR197-3-9GGC	7.93	−53.68	1100	Oppo *et al*.^95^	OC320
KNR197-3-46CDH	7.84	−53.66	947	Oppo *et al*.^95^	OC320
KNR197-3-47CDH	7.84	−53.66	671	Oppo *et al*.^95^	OC320
KNR197-3-53GGC	8.23	−53.23	1272	Oppo *et al*.^95^	OC320
KNR197-3-60GGC	8.44	−52.97	2642	Oppo *et al*.^95^	OC320
KNR197-10-17GGC	36.41	−48.54	5010	Keigwin *et al*.^96^	J + R; OC320; W13; W20
KNR207-2-3GGC	26.14	−44.8	3433	Middleton *et al*.^97^ Middleton *et al*.^98^	J + R; OC320
KNR207-2-6GGC	29.21	−43.23	3018	Middleton *et al*.^98^	J + R
KNR31-GPC5	33.69	−57.61	4583	Keigwin *et al*.^99^ Keigwin *et al*.^100^	J + R; OC320; W13; W20
KNR33-GPC5	33.88	−57.63	4583	Keigwin *et al*.^101^	J + R
M35003-4	12.1	−61.2	1299	Hüls ^102^	J + R; P; OC320; W20
M125_469-3	−10.94	−36.21	1897	Campos *et al*.^103^	OC320

**Table 3:** Continuation of Table 2.

Site	Latitude (°N)	Longitude (°E)	Depth (m)	Isotope data reference	Age models
**ATLANTIC OCEAN**					
MD01-2461	51.75	−12.91	1153	Peck *et al*.^104^	OC320; W20
MD03-2698	38.24	−10.39	4602	Lebreiro *et al*.^105^	W13; W20
MD03-2707	2.5	9.39	1295	Weldeab *et al*.^106^	J + R; OC320; W13; W20
MD07-3076Q	−44.2	−14.2	3770	Walebroeck *et al*.^45^	J + R; P; OC320; W13; W20
MD08-3180	38	−31.13	3050	Repschläger *et al*.^74^	J + R; W13; W20
MD09-3256	−3.55	−35.39	3537	Skinner *et al*.^107^	OC320
MD09-3257	−4.24	−36.35	2344	Skinner *et al*.^107^	OC320
MD13-3455G	35.44	−2.51	319	Fentimen *et al*.^108^ Risebrobakken *et al*.^109^	OC320
MD95-2037	37.09	−32.02	2159	Labeyrie *et al*.^110^	OC320; W13; W20
MD95-2039	40.6	−10.4	3381	Schönfeld *et al*.^111^	J + R; P; W13; W20
MD95-2040	40.6	−9.9	2465	Schönfeld *et al*.^111^	J + R; P; W13; W20
MD95-2042	37.78	−10.17	3146	Hoogakker *et al*.^112^	J + R; W13; W20
MD95-2043	36.14	−2.62	1841	Cacho *et al*.^113^	J + R
MD99-2339	35.89	−7.53	1177	Voelker *et al*.^114^	J + R
MD99-2334	37.8	−10.2	3146	Skinner *et al*.^115^ Skinner *et al*.^107^	O; P; OC320; W13; W20
MD99-2343	40.5	4.03	2391	Sierro *et al*.^116^ Frigola *et al*.^117^	J + R; OC320
MSM05-5-712-1	78.92	6.77	1491	Werner *et al*.^118^	J + R
MSM05-5-712-2	78.92	6.77	1389	Werner *et al*.^118^	J + R
NA87-22	55.5	−14.7	2161	Duplessy *et al*.^119^	J + R; P; OC320; W13; W20
NEAP_04K	61.5	−24.17	1627	Rickaby *et al*.^120^	J + R
OCE205-2-100GGC	26.07	−78.03	1057	Slowey *et al*.^121^ Came *et al*.^94^	J + R; OC320; W13; W20
OCE205-2-103GGC	26.07	−78.06	965	Curry *et al*.^50^	J + R; W13; W20
ODP-108-658	20.75	−18.58	2274	Tiedemann *et al*.^122^	J + R
ODP-162-983	60.4	−23.6	1984	Raymo *et al*.^123^	P; OC320; W13; W20
ODP-162-984	61	−24	1650	Praetorius *et al*.^124^	J + ; P; OC320
ODP-172-1059	31.67	−75.42	2985	Hagen *et al*.^125^	J + R
POS457-905-2	62.69	−14.35	1598	Mirzaloo *et al*.^126^	OC320
POS457-909-2	62.84	−12.99	756	Mirzaloo *et al*.^126^	OC320
PS1243	69.37	−6.55	2177	Bauch *et al*.^127^	J + R; OC320
PS2082-1	−43.22	11.738	4610	Mackensen *et al*.^128^	OC320
PS2498-1	−44.15	−14.23	3783	Mackensen *et al*.^128^	J + R; OC320
PS2561-2	−41.86	28.54	4465	Krueger *et al*.^129^	OC320
RAPiD-10-1P	62.97	−17.59	1237	Thornalley *et al*.^130^	W13; W20
RAPID-12-1K	62.09	−17.82	1938	Thornalley *et al*.^130^	J + R; OC320
RAPiD-15-4P	62.29	−17.13	2133	Thornalley *et al*.^131^	J + R; OC320
RAPiD-17-5-P	61.48	−19.54	2303	Thornalley *et al*.^131^	J + R; W13; W20
RC11-83	−41.6	9.8	4718	Charles *et al*.^132^	J + R; OC320
RC16-119	−27.71	−46.51	1567	Oppo *et al*.^90^	J + R; OC320
RC16-84	−26.71	−43.33	2438	Oppo *et al*.^90^	J + R; OC320
SAN-76	−24.43	−42.28	1682	Toledo *et al*.^133^	OC320
SHAK-03-6K	37.71	−10.49	3729	Skinner *et al*.^107^	OC320
SHAK-14-4G	37.84	−9.72	2063	Skinner *et al*.^107^	OC320
SO164-17-2	24.08	−80.89	954	Bahr *et al*.^134^	J + R
SO75-3-26KL	37.82	−9.5	1099	Zahn *et al*.^135^	J + R; OC320
SO82-5-2	59.19	−30.9	1416	van Krevald *et al*.^136^	J + R; OC320; W13; W20
SU81-18	37.77	−10.18	3135	Duplessy *et al*.^137^	J + R; OC320; W13; W20
SU90-03	40.1	−32	2475	Cortijo *et al*.^138^	P; OC320
SU90-08	43.35	−30.41	3080	Missiaen *et al*.^139^	OC320
SU90-24	61.3	−23	18	Elliot *et al*.^140^	J + R; OC320; W13; W20
SU90-39	52.5	−22	39	Labeyrie ^47^	P
V23-81	54.25	−16.83	2393	Jansen *et al*.^141^	J + R; OC320
V24-253	−26.95	−44.68	2069	Oppo *et al*.^90^	J + R; OC320
V25-59	1.37	−33.48	3824	Sarnthein *et al*.^142^	J + R
V26-176_b	36.048	−72.37	3942	Curry *et al*.^48^	J + R; OC320
V28-14	64.78	−29.57	1855	Curry *et al*.^48^	J + R
V28-122	11.93	−78.68	3623	Oppo *et al*.^143^	OC320
V28-127	11.65	−80.13	1800	Oppo *et al*.^144^	J + R
V29-202	61	−21	2658	Oppo *et al*.^145^	J + R; OC320; W13; W20
V29-204	61.18	−23.02	1849	Curry *et al*.^50^	J + R

**Table 4:** Continuation of Table 3.

Site	Latitude (°N)	Longitude (°E)	Depth (m)	Isotope data reference	Age models
**INDIAN OCEAN**					
AAS9_21	14.51	72.65	1807	Naik *et al*.^146^	O; OC320
FR10-95-GC17	−22.129	113.50	1093	Murgese and De Deckker ^147^ van der Kaars and De Deckker ^148^	OC320
GeoB12615-4	−7.14	39.84	446	Romahn *et al*.^149^	OC313; OC320
GeoB12616-4	−6.98	40.39	1449	Romahn *et al*.^149^	OC313; OC320
M5_3a-422_2	24.39	58.04	2732	Sirocko *et al*.^150^	OC320
MD01-2378	−3.1	121.8	1783	Holbourn *et al*.^151^ Xu *et al*.^152^ Durkop *et al*.^153^	J + R; P; OC320
MD02-2588	−41.33	25.83	2907	Ziegler *et al*.^154^	OC313; OC320; W13; W20
MD02-2589	−43.38	25.25	2660	Molyneux *et al*.^155^	OC313; OC320
MD12-3396Cq	−47.73	86.69	3615	Gottschalk *et al*.^156^	O; OC320
MD77-176	14.5	93.1	1375	Ma *et al*.^157^	O; OC320
MD77-191	7.5	76.7	1254	Ma *et al*.^158^	O
MD77-203	20.70	59.57	2442	Sarnthein *et al*.^142^	OC320
MD84-527	−43.82	51.32	3262	Pichon *et al*.^159^	OC320
MD88-769	−46.07	90.11	3420	Rosenthal *et al*.^160^	OC320
Orgon4-KS8	23.5	59.2	2900	Sirocko *et al*.^161^	P; OC320
RC12-344	12.77	96.07	2140	Naqvi *et al*.^162^	OC313; OC320
SK129-CR2	3	76	3800	Piotrowski *et al*.^163^	OC313; OC320
SK157-GC14	5.18	90.08	3306	Ahmad *et al*.^164^	J + R; OC313
SK157-GC15	7.8	90.25	2855	Raza *et al*.^165^	OC313; OC320
SK157-GC16	8.77	90.3	2920	Raza *et al*.^165^	OC313; OC320
SK157-GC18	11.98	90.02	3069	Raza *et al*.^165^	OC313; OC320
SO236-52-4	3.92	73.14	381.1	Bunzel *et al*.^166^	OC313; OC320
SO42-74KL	14.3	57.3	3212	Sirocko *et al*.^167^	J + R; P; OC320
WIND-28K	−10.15	51.01	4157	McCave *et al*.^168^	J + R; OC313; OC320

**Table 5:** Continuation of Table 4.

Site	Latitude (°N)	Longitude (°E)	Depth (m)	Isotope data reference	Age models
**PACIFIC OCEAN**					
EW0408-26JC	56.96	−136.43	1623	Praetorius *et al*.^169^	M
EW0408-85JC	59.56	−144.15	682	Davies *et al*.^170^	M
EW0408-87JC	58.77	−144.50	3680	Praetorius *et al*.^169^	M
EW9504-02PC	31.25	−117.58	2042	Stott *et al*.^171^	J + R
EW9504-03PC	32.05	−117.58	1299	Stott *et al*.^171^	J + R; OC320
EW9504-04PC	32.04	−118.4	1759	Stott *et al*.^171^	J + R; OC320
EW9504-05PC	32.48	−118.13	1818	Stott *et al*.^171^	J + R; OC320
EW9504-08PC	32.8	−118.8	1442	Stott *et al*.^171^	J + R; OC320
EW9504-09PC	32.95	−119.95	1194	Stott *et al*.^171^	J + R; OC320
EW9504-13PC	36.99	−123.27	2510	Mix *et al*.^172^	M
EW9504-13TC	36.99	−123.27	2510	Mix *et al*.^172^	M
EW9504-14PC	39.39	−124.15	889	This work (Alan Mix)	M
EW9504-17PC	42.24	−125.89	2671	This work (Alan Mix)	M
FR1-97-GC12	−23.57	153.78	990	Bostock *et al*.^173^	J + R; M; OC320
GIK17940-2	20.12	117.38	1727	Wang *et al*.^174^	J + R; OC320
GIK17961-2	8.51	112.33	1795	Wang *et al*.^174^	J + R
H214	−36.93	177.44	2045	Sikes *et al*.^13^	J + R; M; O; OC320
HYIV2015-B9	10.25	112.73	2603	Li *et al*.^175^	OC320
IODP-323-U1339	54.67	−169.98	1867	Cook *et al*.^176^	M
KS15-4-St3PC2	29.46	133.56	2787	This work (Yusuke Okazaki)	OC313; OC320
MD01-2416	51.27	167.72	2317	Gebhardt *et al*.^177^	OC320
MD01-2420	36.06	141.82	2101	Sagawa *et al*.^178^ Okazaki *et al*.^179^	OC313; OC320
MD02-2489	54.39	−148.92	3640	Gebhardt *et al*.^177^	J + R; OC320
MD02-2499	41.68	−124.94	904	Lopes and Mix ^180^	M
MD05-2904	19.45	116.25	2066	Huang *et al*.^181^	OC320
MD06-2986	−43.45	167.9	1477	Ronge *et al*.^182^	J + R; OC320
MD06-2990	−42.31	169.88	943.5	Ronge *et al*.^182^	J + R
MD97-2106	−45.15	146.28	3310	Moy *et al*.^183^	OC320
MD97-2120	−45.53	174.93	1210	Pahnke *et al*.^184^	M; P; OC320
MD97-2121	−40.38	177.99	2314	This work (Elisabeth Sikes)	O
MD97-2138	−1.25	146.23	1900	This work (Alan Mix)	M
MD97-2151	8.7	109.9	1598	Chen *et al*.^185^	P; OC320
MD98-2181	6.3	125.83	2114	Stott *et al*.^186^	J + R; OC320
ME0005-24JC	0.02	−86.46	2941	Dubois *et al*.^187^	M; OC320
ME0005A-27JC	−1.85	−82.79	2203	Kish ^188^	M
ME0005A-43JC	7.86	−83.61	1368	This work (Alan Mix)	M

**Table 6:** Continuation of Table 5.

Site	Latitude (°N)	Longitude (°E)	Depth (m)	Isotope data reference	Age models
**PACIFIC OCEAN**					
ML1208-31BB	4.68	−160.05	2857	Mulitza *et al*.^56^	OC320
ODP-138-846	−3.1	−90.82	3296	Mix *et al*.^189^	J + R; M
ODP-138-849	0.18	−110.52	3839	This work (Alan Mix)	M
ODP-167-1019	41.68	−124.93	980	This work (Alan Mix)	M
ODP-167-1020	41.01	−126.43	3039	This work (Alan Mix)	M
ODP-202-1234	−36.22	−73.68	1015	Heusser *et al*.^190^	M
ODP-202-1238	−1.87	−82.78	2203	This work (Alan Mix)	M
ODP-202-1239	−0.67	−82.08	1414	This work (Alan Mix)	M
ODP-202-1242	7.86	−83.61	1364	This work (Alan Mix)	M
P7	2.60	−83.99	3085	Pedersen *et al*.^191^	J + R; OC320
PAR87A-10	54.36	−148.47	3664	Zahn *et al*.^192^	OC320
PC75-1	−44.24	179.37	967	Shao *et al*.^193^	OC320
PLDS-7G	−3.34	−102.45	3253	Keigwin *et al*.^194^	OC320
PS75-056-1	−55.16	−114.79	3581	Ullermann *et al*.^195^	OC320
PS75-059-2	−54.21	−125.42	3613	Ullermann *et al*.^195^	J + R; OC320
PS75-104-1	−44.77	174.52	835	Ronge *et al*.^196^	O
RC13-110	−0.1	−95.7	3231	Imbrie *et al*.^197^	M; P
RC13-115	−1.65	−104.84	3621	This work (Alan Mix)	M
RR0503-125JPC	−36.2	176.89	2541	Sikes *et al*.^13^	M; O; OC320
RR0503-41JPC	−39.88	177.67	3836	Sikes *et al*.^13^	M; O
RR0503-79JPC	−36.96	176.59	1165	Sikes *et al*.^13^	M; O; OC320
RR0503-83TC/JPC	−36.74	176.64	1627	Sikes *et al*.^13^	M; O
RR0503-87JPC	−37.26	176.64	663	Sikes *et al*.^13^	M; O
RR0503-87TC	−37.26	176.64	663	This work (Alan Mix)	M
RS147-GC07	−45.15	146.28	3300	Sikes *et al*.^13^	M; O; OC320
SCS90-36	17.99	111.49	2050	Huang *et al*.^198^	OC320
SO136-003GC	−42.30	169.88	944	Ronge *et al*.^182^	J + R; OC320
SO201-2-85	57.50	170.41	975	Max *et al*.^199^	J + R; OC320
SO213-2-59-2	−45.83	−116.88	3161	Tapia *et al*.^200^	J + R; OC320
SO213-2-82-1	−45.78	176.60	2066	Ronge *et al*.^182^	J + R; OC320
SO213-2-84-1	−45.12	174.58	972	Ronge *et al*.^182^	J + R; OC320
TR163-25T	−1.65	−88.45	26	Hoogakker *et al*.^201^	OC320
TTN013-18PC	−1.84	−139.71	4354	Murray *et al*.^202^	M
TTN013-72PC	0.11	−139.4	4298	Murray *et al*.^202^	M
V19-27	−0.467	−82.07	1373	Lyle *et al*.^203^	J + R; M; P
V24-109	0.4	158.8	2367	Shackleton *et al*.^204^	P
Vi-37GC	50.42	167.73	3300	Keigwin ^205^	OC320
W8402A-14GC	0.95	−138.95	4287	Jasper *et al*.^206^	M
W8709A-13PC	42.12	−125.75	2712	Lund *et al*.^207^	M; OC320
Y69-106P	2.98	−86.56	2870	Lyle *et al*.^203^	J + R
Y69-71P	0.08	−86.48	2740	Clark *et al*.^208^	M
Y71-9-101P	−6.38	−106.93	3175	This work (Alan Mix)	M
Z2112	−33.53	166.53	2858	Sikes *et al*.^13^	M; O; OC320

**Table 7:** Taxon flags associated with the different benthic foraminifera species included in our data base.

Species	Flag
*Cibicidoides wuellerstorfi*	1
*Cibicidoides kullenbergi*	2
*Cibicidoides lobatulus*	3
*Cibicidoides pachyderma*	4
*Uvigerina spp*.	5
*Cibicidoides mckannai*	6
*Cibicidoides spp*.	7
*Planulina ariminiensis*	8
*Cibicidoides pseudoungerianus*	9
*Cibicidoides teretis*	10
*Cibicidoides mundulus*	11
*Cibicidoides mabahethi*	12

For most sites, the depth-in-core scale is a quantity directly measured in the core. However, some records are based on spliced sections (mainly Ocean Drilling Program (ODP) and Integrated Ocean Drilling Program/International Ocean Discovery Program (IODP) sites) of several nearby cores to generate a composite with a corresponding composite depth to define the seafloor referenced depth scale for the site. When available, these depth models are documented in the database, accompanied by archival depths that correspond to the original depth within each cored interval.

To have a measure of the uncertainty in the timing of deglacial shifts in isotope time series, we include as many published age models associated with the data series as attainable. Only those age models that include information about how they were calculated are included. Age models were either obtained from original publications and recent syntheses, or generated for this work. We include age models from three published compilations, which focus mostly on Atlantic sites:

- From Peterson *et al*.^19^ we include age models for 48 sites, calculated using benthic foraminiferal *δ*^18^O values combined with radiocarbon-based age models^14^. These age models are referred to as P hereafter.
- From Waelbroeck *et al*.^12^ we include Undatable software age models^20^. They were calculated from planktic foraminiferal calibrated accelerator mass spectrometry (AMS) radiocarbon dates in low- and mid-latitude sites. In areas of large changes in surface reservoir ages, they were calculated using a combination of radiocarbon dates and alignment tie points between sea surface temperature or magnetic property records to ice core records. We include age models for 44 sites from the original publication, with radiocarbon data calibrated to the IntCal13^21^ curve, and age models for 48 sites from an update using the IntCal20^22^ calibration curve. These age models are referred to as W13 and W20, respectively, hereafter.
- From compilations by Jonkers *et al*.^23^ and Repschläger *et al*.^18^ we include age models from 151 sites (referred to as J + R hereafter). We combine these two compilations because they share Atlantic sites and methodologies. Most age models are based on AMS radiocarbon dates on planktic foraminifera using the software BACON^24^ version 2.3.9.1 within the data management toolbox PaleoDataView^25^ and calibrated to the IntCal13^21^ curve. Some additional age models in Repschläger *et al*.^18^ were calculated using benthic foraminiferal *δ*^18^O stratigraphy or using automated alignment with a stacking method described in Lee *et al*.^26^.

The database includes several sets of age models calculated for this publication:

- 41 new age models for Pacific sites calculated based on benthic foraminiferal *δ*^18^O stratigraphy aligned to the LR04 stack^27^ between the LGM and the early Holocene.
- 17 new age models calculated from AMS radiocarbon dates on planktic foraminifera calibrated to the IntCal13^21^ curve with the software BACON^24^ version 2.3.9.1. All parameters are recorded in the database as age model text files. These age models were calculated before the release of the IntCal20^22^ calibration curve.
- 211 new age models calculated using the software BACON^24^ version 2.3.9.1 within the data management toolbox PaleoDataView^25^. Radiocarbon data were calibrated using the IntCal20 calibration curve^22^. Prior to calibration and BACON age modeling, a local reservoir age simulated with the *Large Scale Geostrophic ocean general circulation model*^28^ over the last 55 ky^29^ was subtracted. To produce local time series of the total radiocarbon age versus reservoir age, we added the modelled reservoir ages to the IntCal20 radiocarbon ages (by associating the modeled and IntCal20 calendar ages). For each measured radiocarbon age we then selected the corresponding local reservoir age. Specifically, the surface (0–50 m) reservoir age range corresponding to the measured radiocarbon age range from the nearest gridbox in the simulated data were extracted. The downcore age model and its uncertainties is based on 1000 BACON age-depth realizations. All parameters are recorded in the database as age model text files. The sites in this age model ensemble include the 17 sites for which we calculated age models with IntCal13 calibration as described above.

## Data Records

### Data Availability

The database was developed by the OC3 community, following the FAIR (Findability, Accessibility, Interoparability, Reusability) guiding principles for scientific data management and stewardship^30^. Conforming to the accessibility principle (the “A”) of the FAIR data standard, the database has been stored in the public repository Zenodo^31^. This repository allows updates on the database after publication. Future additions of new sites and age models will be uploaded by the OC3 members.

### Database description

**Fig. 1:**
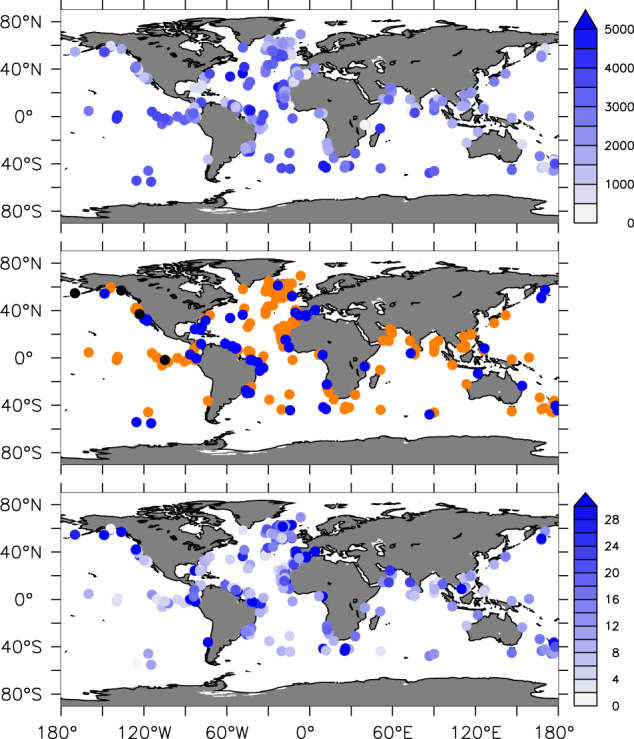
(Top) Positions and depths (in m) of all sites included in our database. (Middle) Isotope data species codes: (orange) *Cibicidoides wuellerstorfi*, (blue) other *Cibicidoides*, (black) other benthic foraminifera. (Bottom) Number of data points at each site in the 23-15 ky BP time interval.

**Fig. 2:**
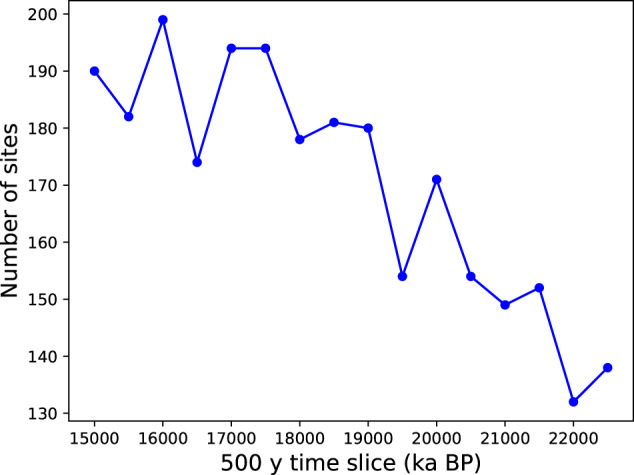
Number of sites per 500 y time slice in our data base.

Sites included in Version 1.0 of the OC3 database are listed in Tables 1–6, with citations for isotope data and age models. They come from the global ocean and a water depth range between 200 and 5000 m (Fig. 1, top). 98% of sites report stable isotope data from *Cibicidoides spp*., and 74% correspond to *Cibicidoides wuellerstorfi* (Fig. 1, middle). We include some sites that report unpublished data obtained from other species, mostly *Uvigerina spp*. The number of isotope measurements at each site (Fig. 1, bottom) for 23-15 ky BP has a mean of 16 and a median of 12 data points available per record. 84% of sites have a time resolution of at least 1 ky for either the 23-19 or 19-15 ky BP time slices. The remaining sites were included because they either have 1 ky or higher resolution for the subsequent 15-11 ky BP time slice, or because they present new, unpublished data (see Tables 1–6). We include in Zenodo a table with the number of data points for the 23-19, 19-15, and 15-11 ky BP time slices at each site^31^. Users may use that tables or software tools that accompany this publication^31^ to discern, based on temporal resolution and region, which sites to include in their analyses. Binning the data into 500-year time slices between 23 and 15 ky BP, yields 130 to 200 coring sites per time slice (Fig. 2), with a higher number in the ED. Geographically, 63% of sites correspond to the Atlantic, 28% are from the Pacific, and 9% correspond to the Indian Ocean. 12% sites lie in the Southern Ocean (south of 35 °S).

### Database structure

**Fig. 3:**
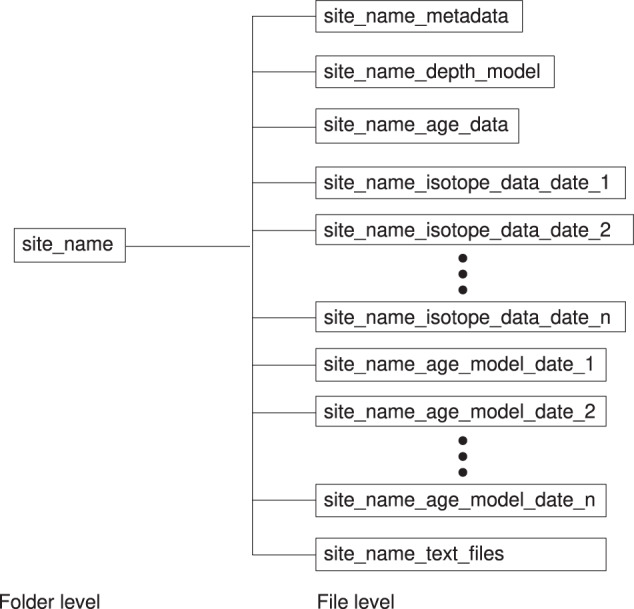
Diagram of a general OC3 site folder, with its file structure as described in the text.

The database is organized in different folders, each named after and corresponding to a specific coring site. The folders contain comma separated value (csv) files (Fig. 3). The file format choice makes the files easily machine-readable on computers with different operating systems, conforming to the interoperability principle (the “I”) of the FAIR data standard. It also makes them human-readable, which facilitates access and editing. Each site folder contains at least one of each of the following file types:

- A metadata file, with ocean basin, site name, latitude, longitude, and seafloor depth.
- A depth model file with depth scale information.
- An age data file, with measured age constraints (e.g., radiocarbon) and/or tie points information, including type of age constraints and references.
- Isotope data files, with *δ*^13^C and/or *δ*^18^O data on a depth scale, and measurement methodology, taxon, and reference. There can be more than one isotope data file, each corresponding to different taxa, or as new data is added to the site. The different isotope files are identified in their names with dates of addition to the database in year-month-day (yyyymmdd) format, author name, and/or taxon name.
- Age model files, with depth scale and age determinations, and information on age model type and source. There can be more than one age model file, each corresponding to a different age model. The different age model files are identified in their names with dates of addition to the database in year-month-day (yyyymmdd) format and/or author name.

The csv files are accompanied by unformatted text files where additional information is documented. All files are identified with the same site name as in the database, to conform the findability principle (the “F”) of the FAIR data standard.

In addition to the raw data and age models, we include the reference and when available, name of the laboratory and methodology followed for analysis. For radiocarbon-based age models calculated with the software BACON, we include all parameters used in the calculation in separate age model text files included within each of the site folders. This aims to fulfill the reusability principle (the “R”) of the FAIR data standard. Columns are left blank when the information is not available, but they could be filled in with new version releases and new contributions. The data type and format of each column in the csv files is specified as follows. Missing data are indicated with a blank column. Columns with the “Notes” label in their name are to be used by operators to add unformatted information that they consider relevant. For stable isotopes the units used are permil, in terms of Vienna PDB (VPDB).

- site_metadata.csv
  Ocean: Pacific, Indian, Atlantic (includes Arctic and Mediterranean).
  Sea: A more specific region, if it corresponds, e.g., South China Sea
  Site: Site name. Corresponding to the name that appears in the files and folder names. For Deep Sea Drilling Project (DSDP)/ODP/IODP sites we use DSDP/ODP/IODP-leg/expedition-site as name convention.
  Latitude (degN): Latitude, with the highest precision possible. Between −90 and 90 °N)
  Longitude (degE): Longitude, with the highest precision possible. Between −180 and 180 °E
  Site Depth (m): Depth of the sea floor below modern mean sea level, with the highest precision possible, in negative numbers.
- site_depth_model.csv
  Site: Site as in metadata file.
  sample_label: Label of individual sample from original publication, if available.
  hole_label: Label for holes in the site, for sites that include more than one hole.
  section_label: Label of section in the core.
  published_archival_depth (m): In cases where only one core is sampled at each site, this usually coincides with the reported depth in core of the original publication. For sites with more than one core (e.g., IODP sites), it is defined as the value assigned by the estimated depth of the bottom of the drill string below the sea floor, plus the sum of the depths in sections in the cores shallower than the section being analyzed.
  current_depth_model (m): It coincides with the archival depth in sites where only one core is sampled. For sites with more than one core (e.g., IODP sites), the depth model transforms archival depths into true sample depths, considering processes such as compression/expansion during the coring process.
  current_depth_model_note: Any important information on the depth model.
  DEPTH(mid) (m): As defined for IODP cores^32^.
  MBSF(mid) (m): Meters below sea floor, as defined for IODP cores^32^.
  MCD(mid) (m): Meters composite depth, as defined for IODP cores^32^.
  CCSF(mid) (m): Core composite depth below sea floor, as defined for IODP cores^32^.
  depth_model_1 (m): Spaces to include older depth models. This column is usually filled with a copy of the published_archival_depth (m) column.
  depth_model_note_1: Any important information on depth_model_1.
  older_depth_model_2 (m): Spaces to include older depth models. More columns of this kind may be added if needed.
  older_depth_model_note_2: Any important information on older_depth_model_2.
- site_isotope_data_yyyymmdd.csv
  Site: Site as in metadata file.
  Sample Label: Label of individual sample.
  archival_depth (m): Archival depth at which data were taken.
  d13C (permil): Benthic foraminiferal *δ*^13^C values without any vital effect corrections.
  d18O (permil): Benthic foraminiferal *δ*^18^O values without any vital effect corrections.
  d13C_corrected (permil): Benthic foraminiferal *δ*^13^C values with vital effect corrections.
  d18O_corrected (permil): Benthic foraminiferal *δ*^18^O values with vital effect corrections.
  Number of shells: Number of shells measured.
  Minimum mesh size (um): Minimum mesh size used for (dry) sample sieving prior to picking.
  Maximum mesh size (um): Maximum mesh size used for (dry) sample sieving prior to picking.
  Taxon: Taxon of sample, e.g., *Cibicidoides wuellerstorfi*.
  Taxon_flag: A number that identifies the species. See Table 7 for the list of taxon flags.
  Taxon_note: A note on the taxon.
  Taxon_note2: Space for notes on taxon or methodology.
  Taxon_note3: Space for notes on taxon or methodology.
  Additional_note: Note on methodology.
  Publication source: Publication from where data were obtained.
  Original reference: Original publication associated with the data.
  File name: File name in original repository.
  Data source: Publication where data is found. Usually a Digital Object Identifier (DOI).
  Quality control: 1 means that the data has been quality controlled as described in the data acquisition section. 0 means that the data were defined as an outlier or bad data in the quality control process.
- site_age_data.csv
  Site: Site as in metadata file.
  Sample label: Label of individual sample.
  sample_depth: Depth in core (meters below the sea floor) for the sample, in meters.
  technique: Method used to calibrate age data into calendar age.
  lab. code: Identifying code of the laboratory where the age data were taken.
  species/material: Species or type of material used for age measurements.
  radiocarbon_age (y): Measured conventional radiocarbon ages (using Libby’s half-life).
  radiocarbon_age_error_plus (y): Uncertainty of the radiocarbon dates in the positive direction.
  radiocarbon_age_error_minus (y): Uncertainty of the radiocarbon dates in the negative direction.
  reservoir_age (y): Estimated reservoir age used to calculate the calendar age
  reservoir_age_error_plus (y): Uncertainty of the estimated reservoir age in the positive direction.
  reservoir_age_error_minus (y): Uncertainty of the estimated reservoir age in the negative direction.
  calendar_age (y BP): Calibrated age.
  calendar_age_error_plus (y BP): Uncertainty of the calibration in the positive direction.
  calendar_age_error_minus (y BP): Uncertainty of the calibration in the negative direction.
  calibration curve: Calibration curve used to calculate calendar ages (e.g., IntCal13; IntCal20).
  note1: Unformatted information considered relevant.
  note2: Unformatted information considered relevant.
  original reference: Reference on the age data and/or the calibrated age.
  data doi: age data DOI and/or reference.
- site_age_model_yyyymmdd.csv

Site: Site as in metadata file.

Sample Label: Label of individual sample.

age_model_depth (m): Depths at which the age model is calculated.

age_model (y BP): Modeled calendar age.

age_model_sigma_plus (y BP): Uncertainty of modeled age in the positive direction.

age_model_sigma_minus (y BP): Uncertainty of modeled age in the negative direction.

upper_95_percent (y BP): 95% confidence level of modeled age in the positive direction.

lower_95_percent (y BP): 95% confidence level of modeled age in the negative direction.

age_flag: Number flag indicating age model method. See Table 8.

**Table 8:** Age model flags associated with different methodologies included in the data base.

Age model type	Flag
Ash layers	1
^14^C plateau tuning	2
^14^C accelerator mass spectrometry dates	3
Tuned age model using benthic *δ*^18^ O data with benthic stacks	4
Tuned age model using *δ*^18^ O aligment with high resolution land archives (e.g. ice cores)	5
Biostratigraphy	6

age_model_note: Any note on the age model.

age_model_collection.

quality control: 1 means that the data has been quality controlled as described in the Data acquisition section.

All file names begin with a string referring to the core site that matches the site name in the metadata files. Isotope data and age model files also include a date in their names, which corresponds to the date at which the information was added to the database, and it is written in yyyymmdd (year-month-day) format. If more than one isotope data and/or age model is available for a particular site, separate files with different dates are created for each one. For sites that include isotope data and/or age models from other syntheses, additional isotope data, age model, and depth model files are included in the corresponding folders, with a distinctive string added to their names. In cases where more than one species was reported for a site, we keep the isotope data and age model associated with each species in separate files, with the species specified in the file names. The name structure and use of csv files in the database allows the user to make specific updates. New isotope data and age models can be easily added, using the date format described above.

## Technical Validation

### Time slice comparison

**Fig. 4:**
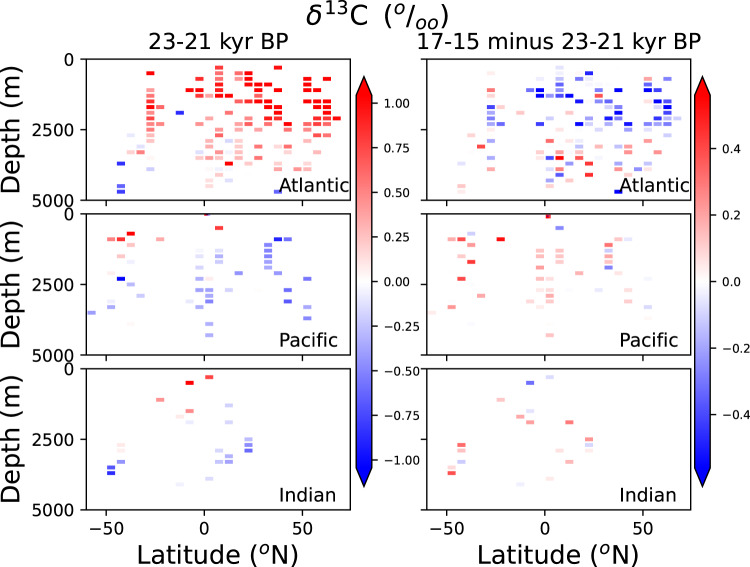
(Left) Zonally-collapsed *Cibicidoides δ*^13^ C values from our database for an LGM time slice (21-19 ky BP). (Right) *Cibicidoides δ*^13^ C difference between a deglacial time slice (17-15 ky BP) and the LGM. In order to calculate differences between the time slices, all data were binned into a latitude-depth grid of 5°×200 m resolution. The most recent age model available at each site was used to make this plot. The lengths of the time slices were chosen such that both were 2000 y long.

Despite its sparsity, the coverage of the database resolves the general structure of deep water masses in depth-latitude plots (Fig. 4). During the LGM, the North Atlantic shows high benthic foraminiferal *δ*^13^C values in the North Atlantic above 2500 m, associated to the glacial equivalent North Atlantic Deep Water^9^ (NADW). Deeper Atlantic waters exhibit lower *δ*^13^C values related with a mixture of glacial NADW and Antarctic Bottom Water. In the Pacific, *δ*^13^C-depleted Pacific Deep Water can be distinguished, as well as shallower, *δ*^13^C-enriched waters in the Southern Ocean associated with the transport of Antarctic Intermediate Water.

In the Atlantic, compared with the LGM, deglacial benthic foraminiferal *δ*^13^C values from the 17-15 ky time slice (Fig. 4, right) is lower in northern-component waters (above 2500 m) and higher in most sites in regions of southern-component waters. This is in agreement with previous reconstructions^19,33,34^, and consistent with Atlantic Meridional Overturning Circulation shallowing and accumulation of respired carbon in deep waters^35^. Benthic foraminiferal *δ*^13^C is also higher in the Pacific and Indian Oceans in the 17-15 ky time slice time slice compared with the LGM.

**Fig. 5:**
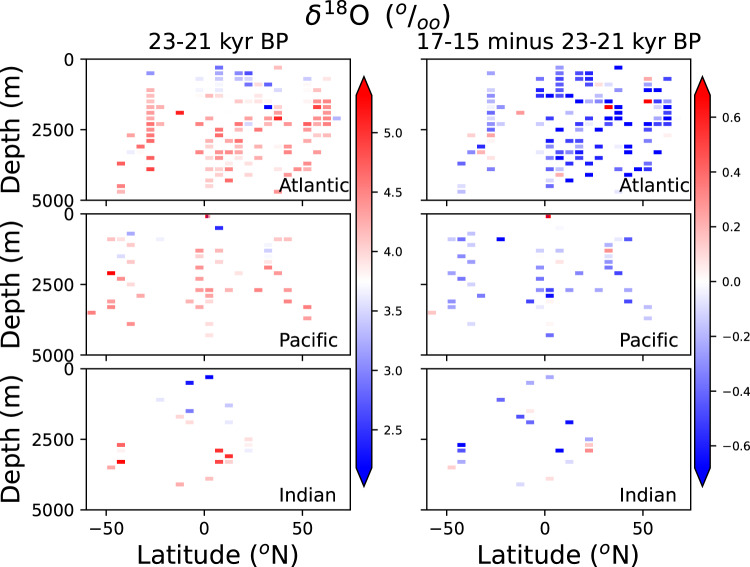
As Fig. 4, but for *Cibicidoides δ*^18^ O data.

Concerning benthic foraminiferal *δ*^18^O values, inter-laboratory calibration offsets of several tenths of a per mil complicate the analysis of anomalies^36,37^, proving it difficult to have a quantitative measure of LGM-deglacial changes. However, a decrease is observed in most regions between the 17-15 ky time interval and the LGM (Fig. 5). This decrease reflects deglacial changes in temperature and *δ*^18^O values of deep waters^38^.

### Age model comparisons

The OC3 database includes sites with more than one age model (Tables 1–6), allowing an evaluation of the sensitivity of the reconstructed time evolution of benthic foraminiferal *δ*^13^C and *δ*^18^O values with respect to different age models. Such analysis gives insights into the bias associated with age model uncertainties and enables us to investigate the robustness of leads and lags between deglacial stable isotope records.

**Fig. 6:**
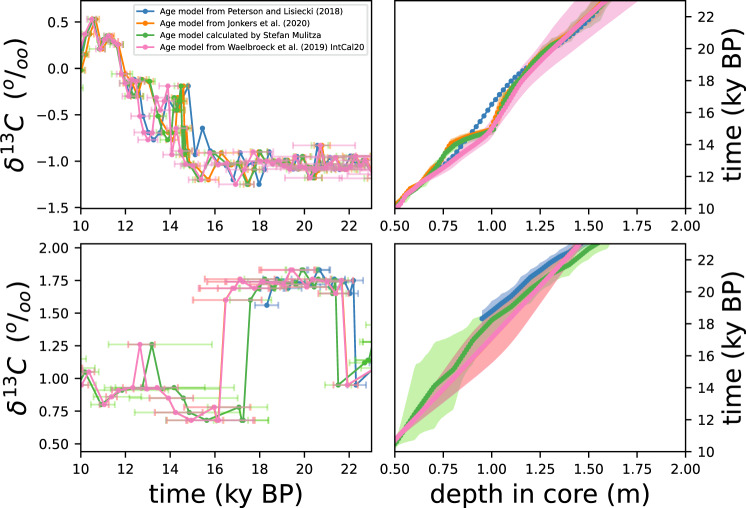
(Left) Time series of *Cibicidoides δ*^13^ C values calculated with age models from different compilations, as indicated. Age uncertainty bars are included. (Right) Age models as a function of depth in core. Shadings correspond to the reported age uncertainties, based on 95 % confidence intervals. Two example sites from the database are displayed: (top) MD07-3076Q and (bottom) SO82-5-2. Plots for the rest of the sites are included in the Zenodo repository^31^. Age models labeled as “calculated by Stefan Mulitza” were calculated for this work from radiocaron dates with the IntCal20 calibration curve, as explained in the Methods section (OC320 age models in Tables 1–6).

We include in the Zenodo repository^31^ plots of benthic foraminiferal *δ*^13^C and *δ*^18^O values versus age of all sites. The lags between age models are not constant through the LGM and ED (e.g., South Atlantic site MD07-3076Q in Fig. 6) with lags generally comprised between 0 and 1 ky. Even for sites where lags of the order of 2 ky exist (e.g., North Atlanitc site SO82-5-2, Fig. 6), there is overlap among the uncertainty intervals of the age models, meaning that differences in timing are likely smaller than the uncertainties of the respective age estimates.

**Fig. 7:**
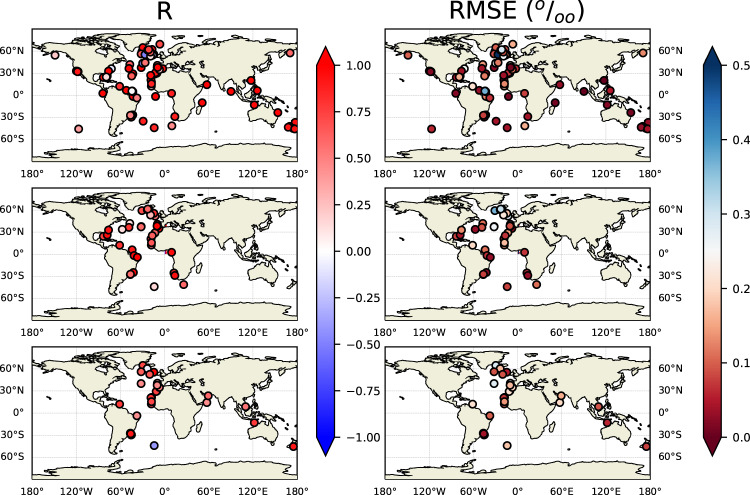
Map distribution of correlation coefficient *R* and *RMSE* of *Cibicidoides δ*^13^ C from OC3 sites between 23 and 15 ky BP calculated with the age models from this work (OC320 in Tables 1–6) and (top) J + R; (middle) W20; (bottom) P age models.

To further assess the impacts of age model on the data assessment, we calculated the correlation coefficient *R* and root mean square error *RMSE* at each site, between the benthic foraminiferal (*Cibicidoides*) *δ*^13^C time series generated for this work from ^14^C-calibrated age models (labeled as OC320 in Tables 1–6) and with other age models, namely the J + R, W20, and P (previous compilations). The time window chosen for this analysis is 23-15 ky BP, and mostly Atlantic Ocean sites are used, since most sites with multiple age models are situated there (Fig. 7). To allow the calculation of correlations and *RMSE*, all data were linearly interpolated to a regular age grid with a 500 y time step. Other time steps were trialed (100 and 1000 y), yielding no different results. Correlation coefficients have values higher than 0.60 in 73% and 54% of the sites for the comparison of OC320 with the W20 or P age models, respectively. The comparison of *Cibicidoides δ*^13^C time series generated with the OC320 and J + R age models yields correlation coefficients higher than 0.60 for 75% of the sites, highlighting the high compatibility of ^14^C age models that use the same methodology. Discrepancies in several North Atlantic sites, that lead to low and even negative correlations between time series (Fig. 7, left), are due to surface reservoir age differences among age model approaches. The comparison among time series calculated with either of the age models yields *RMSE* values lower than 0.3 permil in 90% of the cases (red circles in Fig. 7, right panels). The discrepancies among time series of *Cibicidoides δ*^13^C values associated with the use of different age model approaches are thus generally lower than estimates of LGM-Holocene changes in benthic foraminiferal *δ*^13^C values (0.38 permil^39^).

**Fig. 8:**
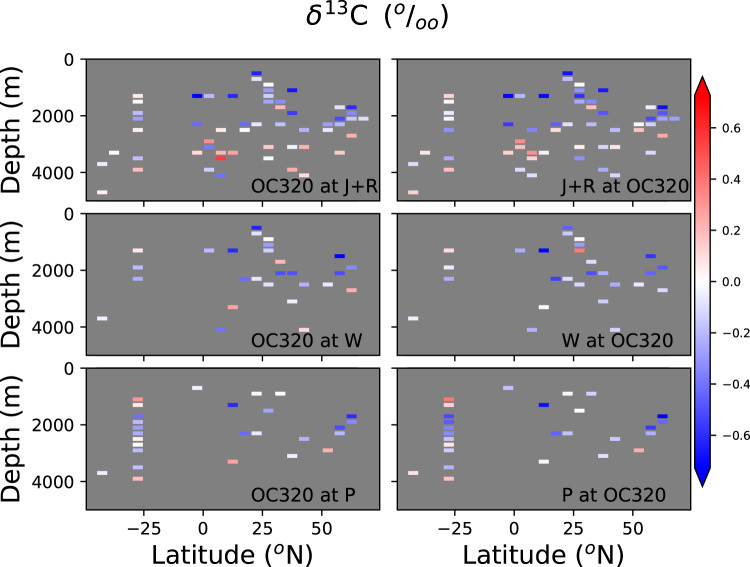
Comparison of latitude-depth Atlantic sections calculated for the difference between the 21-19 and 17-15 ky BP time slices. First row of plots: *Cibicidoides δ*^13^ C time slice calculated with OC320 (as in Tables 1–6) age models at sites where both OC320 and J + R age models are available. Left(right) plot shows the time slice calculated with OC320(J + R) age models. Second(third) row of plots: Same as top plots but for OC320 and W20(P) age models. Data were binned to the same grid than in Fig. 3. Correlation coefficients and *RMSE* are (OC320 and J + R comparison) 0.83, 0.20 permil; (OC320 and W20 comparison) 0.75, 0.19 permil; (OC320 and P comparisons) 0.90, 0.13 permil, respectively.

**Fig. 9:**
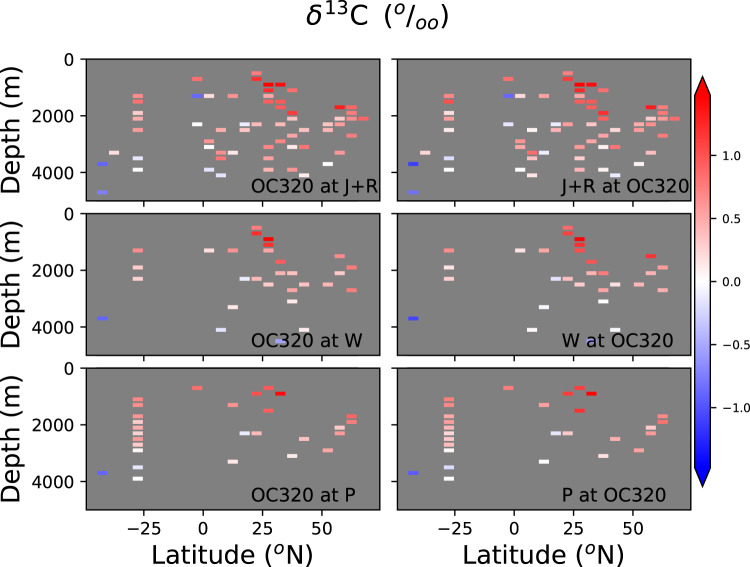
Comparison of latitude-depth Atlantic sections calculated for 17-15 ky BP time slice. First row of plots: *Cibicidoides δ*^13^ C time slice calculated with OC320 (as in Tables 1–6) age models at sites where both OC320 and J + R age models are available. Left(right) plot shows the time slice calculated with OC320(J + R) age models. Second(third) row of plots: Same as top plots but for OC320 and W20(P) age models. Data were binned to the same grid than in Fig. 8. Correlation coefficients and normalized *RMSE* are (OC320 and J + R comparison) 0.93, 0.18 permil; (OC320 and W20 comparison) 0.93, 0.19 permil; (OC320 and P comparisons) 0.97, 0.11 permil, respectively.

Another approach to assess age model uncertainty is to compare time slices generated with the same data, but with different age model approaches. We compare sites with radiocarbon age models calculated for this publication (OC320 in Tables 1–6) and other age model compilations. We calculated at each site the *Cibicidoides δ*^13^C difference between the 21-19 and 17-15 ky BP time slices (Fig. 8). Due to the scarcity of records in other basins, the analysis is limited to the Atlantic Ocean. The *Cibicidoides δ*^13^C time slice difference calculated using OC320 age models is similar in spatial structure to the time slice differences calculated using J + R, W20, and P age models (comparison of left- and right-side plots in Fig. 8). Correlation coefficients are 0.83, 0.75, and 0.90, respectively. This reflects a high agreement in the direction of deglacial changes in *δ*^13^C values, irrespective of which age model is used. The corresponding *RMSE*′s are 0.20, 0.19, and 0.13 permil, which is of the same order of magnitude as the differences in *δ*^13^C values between the two time slices at each individual site (Fig. 8). This indicates that the resulting magnitude of *Cibicidoides δ*^13^C changes between time slices may differ considerably when using different age model approaches. We repeated the analysis for the single 17-15 ky BP time slice, without calculating a time slice difference (Fig. 9). In that case we get correlation coefficients higher than 0.9 for the three *Cibicidoides δ*^13^C time slice comparisons, and *RMSE*′s lower than 0.20 permil. The result reflects that *Cibicidoides δ*^13^C in single time slices may be less dependent on the age model approach than the difference between *Cibicidoides δ*^13^C values from different time slices.

The above analyses illustrate that the OC3 database coverage is sufficient to resolve deep ocean water mass features through time. The number of sites in the Pacific and Indian Oceans is still considerably lower than in the Atlantic Ocean, and future versions of the database will focus on improving the coverage for those basins. An analysis of stable isotope distributions through the LGM and ED, whose time dimension were calculated from different age model approaches, shows that the direction of changes may be captured, irrespective of the age model approach used, but the magnitude of those changes differs among age model approaches. The database features allow to construct a four-dimensional picture of stable carbon and oxygen isotopes through the LGM and last deglacial periods. The included software tools^31^ allow quick calculations and the selection of sites for data analysis or model-data comparisons.

## Usage Notes

The choice of csv format for the OC3 database allows accessibility from a wide variety of computer software, and very light computational needs. In order to facilitate analysis, we have created a number of python programming language scripts that perform tasks for users. Because the scripts are equipped with simple user interfaces, no knowledge of python is required.

The python scripts are included in the repository Zenodo, in the same location of the dataset^31^. They are simultaneously compiled and run by entering, in the command line (Windows systems) or terminal (UNIX systems), “python scriptname.py”, where scriptname refers to the name of the chosen python script. The minimum python version required is 3.6. The scripts run locally. In order to retrieve OC3 data, the entire or parts of the OC3 database needs to be downloaded to the local system. In order to run, the scripts need a number of python packages to be installed. The packages needed for each script are listed in the repository^31^.

The scripts provided for analyzing the OC3 database are as follows:

- list_positions.py: This script retrieves the position and site name metadata of a region of interest (defined by longitude, latitude and depth ranges) and lists them in a single csv file. This allows users to quickly visualize the position and basin information of all sites in a chosen region.
- time_series_d13c.py and time_series_d18o.py: These scripts retrieve the data and age models from the OC3 database location and create time series plots (encapsulated postscript (eps) files) of benthic foraminiferal *δ*^13^C and *δ*^18^O values, respectively, with all age models available for each of the sites. The name of the site and the benthic foraminifera species are displayed in the time series images. Age model uncertainties are displayed as error bars when available.
- merge_cores_files_database.py: This script grabs the isotope data from the OC3 location, and lets the user choose one of the available age models to linearly interpolate to the isotope data’s depth-in-core scale. Once the age model is chosen, the script generates a folder of merged csv files with position, age, isotope data, and taxon information for each site. The number of rows of all columns in each generated file is the same, in order to facilitate access with any data analysis software. The following python scripts included with the database make use of the merged csv files generated with this scirpt:
  - list_time_resolution.py: This script lists the number of data points at each site inside a predefined time slice. The result is saved in a csv file.
  - time_slice.py: This script lets the user define a taxon group (*Cibicidoides wuellerstorfi*, any *Cibicidoides*, or all taxa), a time interval, and a region of interest (defined by longitude, latitude and depth ranges), and calculates the time mean of the benthic foraminiferal *δ*^13^C and *δ*^18^O data for all sites that include data in the defined time interval and region. The result is saved in a csv file, and plotted in longitude-latitude, latitude-depth, and longitude-depth two dimensional scatter plots. The images are saved as eps files.
  - compare_time_slices.py: This script lets the user define a taxon group as in the previous script and two time intervals. It plots, in latitude-depth sections for each basin, the benthic foraminiferal *δ*^13^C or *δ*^18^O data from the first time slice (left panels), and the benthic foraminiferal *δ*^13^C or *δ*^18^O difference between the second and first time slices (right panels). The images are saved as eps files. In order to calculate the differences and visualize, the scripts bins the data positions into a regular 5°×200 m grid.

For authors who are not familiar with running python scripts, we also include in Zenodo^31^ merged files (in csv format) that contain metadata, depth, age model, and isotope data for all sites. We include one merged file for each of the age model groups available.

## Data Availability

All code used to generate the figures and analysis of this paper is available in the Zenodo repository^31^.
